# Flexible Glass-Based Hybrid Nanofluidic Device to Enable the Active Regulation of Single-Molecule Flows

**DOI:** 10.1021/acs.nanolett.2c04807

**Published:** 2023-03-07

**Authors:** Hiroto Kawagishi, Shun-ichi Funano, Yo Tanaka, Yan Xu

**Affiliations:** † Department of Chemical Engineering, Graduate School of Engineering, Osaka Metropolitan University, 1-2, Gakuen-cho, Naka-ku, Sakai, Osaka 599-8570, Japan; § Center for Biosystems Dynamics Research, RIKEN, 1-3 Yamadaoka, Suita, Osaka 565-0871, Japan; ∥ Japan Science and Technology Agency (JST), PRESTO, 4-1-8 Honcho, Kawaguchi, Saitama 332-0012, Japan; ⊥ Japan Science and Technology Agency (JST), CREST, 4-1-8 Honcho, Kawaguchi, Saitama 332-0012, Japan

**Keywords:** nanofluidic channels, valves, single molecules, detection, dynamics, nanoconfinement effects

## Abstract

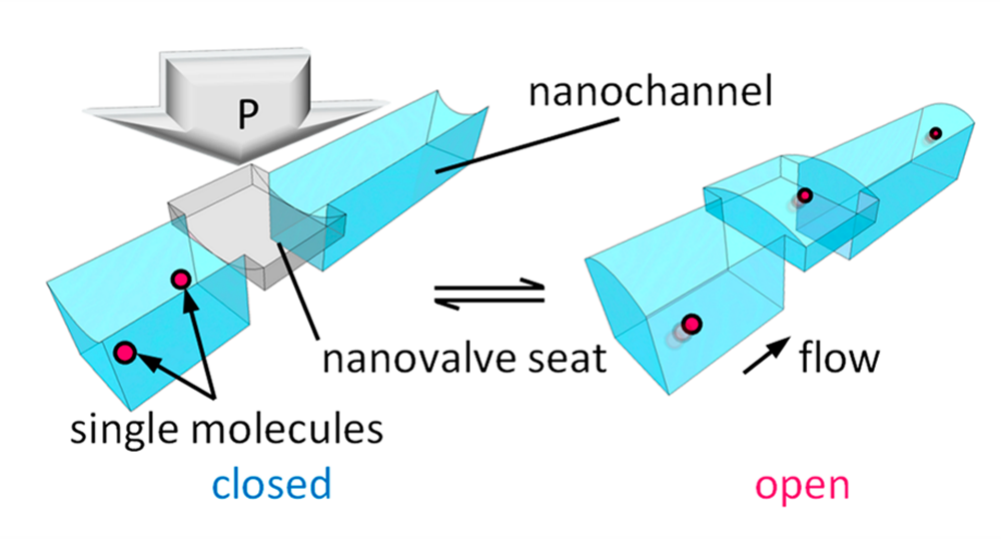

Single-molecule studies offer deep insights into the essence of chemistry, biology, and materials science. Despite significant advances in single-molecule experiments, the precise regulation of the flow of single small molecules remains a formidable challenge. Herein, we present a flexible glass-based hybrid nanofluidic device that can precisely block, open, and direct the flow of single small molecules in nanochannels. Additionally, this approach allows for real-time tracking of regulated single small molecules in nanofluidic conditions. Therefore, the dynamic behaviors of single small molecules confined in different nanofluidic conditions with varied spatial restrictions are clarified. Our device and approach provide a nanofluidic platform and mechanism that enable single-molecule studies and applications in actively regulated fluidic conditions, thus opening avenues for understanding the original behavior of individual molecules in their natural forms and the development of single-molecule regulated chemical and biological processes in the future.

Single-molecule studies have introduced new fields in science and engineering by revealing the stochastic and heterogeneous dynamics of individual molecules, which cannot be obtained by ensemble-averaged measurements, thereby providing comprehensive insights into the essence of chemistry, biology, and material sciences.^1−4^ Sophisticated methodologies, such as immobilization,^5^ trapping,^6^ and confinement^7^ have been developed to better understand the behavior of a single molecule in a solution. However, identifying and precisely regulating the flows of single small molecules is still a formidable hurdle to understanding the actual behaviors of individual molecules and developing applications, such as single-molecule-based sensors, reactors, processors, and computation. This is due to the lack of a platform and methodology that allows for both the active regulation and real-time observation of single small molecules in fluidic conditions, which are the true major forms of the existence of molecules. Here, we focus on small molecules because, in comparison with currently well-studied biomacromolecules, small molecules are universal objects but largely unexplored in single-molecule studies due to their ultrasmall dimensions.

To resolve this challenge, we focus on chip-based nanofluidic devices (hereafter referred to as “nanofluidic devices”), which are solid-state devices containing in-plane nanochannels with a precisely controlled geometry.^4,8−13^ In comparison with popular microfluidic devices, nanofluidic devices are at a nascent stage but possess great potential owing to their special phenomena and nanoscale effects.^14−20^ In particular, owing to the ultrasmall volumes of the nanochannels, nanofluidic devices can confine single molecules at high concentrations ranging from nM to μM, which is the concentration range of reactant molecules in most chemical syntheses and biomolecules in a single cell.^13,21,22^ In addition, owing to their planar, transparent, in-plane, and solid-state characteristics, nanofluidic devices can easily be coupled with a variety of microscopes and exhibit flexibility superior to that of other nanofluidic geometries, such as silicon-based nanofunnels,^23^ carbon nanotubes,^24^ nanopores,^25^ nanopipettes,^26^ nanoporous polymer membranes,^27^ and two-dimensional material membranes.^28^ Hence, nanofluidic devices have been used to manipulate and simultaneously visualize single biomacromolecules, such as DNA^29^ and proteins.^30^ These characteristics suggest that nanofluidic devices hold promise as tools for detecting and regulating single small molecules in fluidic conditions. Although a few nanofluidic valves, whether passive^9^ or active,^33,35^ and whether used with indirect^33^ (i.e., outside of nanochannels) or direct^9,35^ (i.e., inside of nanochannels) methods, demonstrate the ability to regulate net flows in nanochannels, the development of nanofluidic devices with direct, active valving functions capable of precisely blocking, opening, and directing flows of single small molecules has not been explored.

In this study, we regulate the flows of single small molecules using a hybrid nanofluidic device comprising a flexible glass part and a hard glass part with composite nanochannels having nano-in-nano structures as nanovalve seats, which together function as direct and active nanovalves in tiny nanochannels (Figure 1a, b). A reversible nanometer-scale mechanical deformation can be actuated in the flexible glass part, owing to its high mechanical flexibility, by exerting external pressure. Upon reversible nanometer-scale mechanical deformation, the narrow, shallow nanospace precisely formed above the nanovalve seat can be blocked by adjusting the external liquid pressure in an adjacent chamber of working liquid from a bulk vial (Figure 1c, d). Thus, a high-pressure-resistant, reversible valving function in the nanochannels is achieved, enabling the precise blocking, opening, and directing of flows of single small molecules. Our nanofluidic device and approach enable single-molecule studies in actively regulated fluidic conditions, thus opening the way to elucidate the real behaviors of individual molecules in their original forms and exploit single-molecule regulated chemical and biological processes.

**Figure 1:**
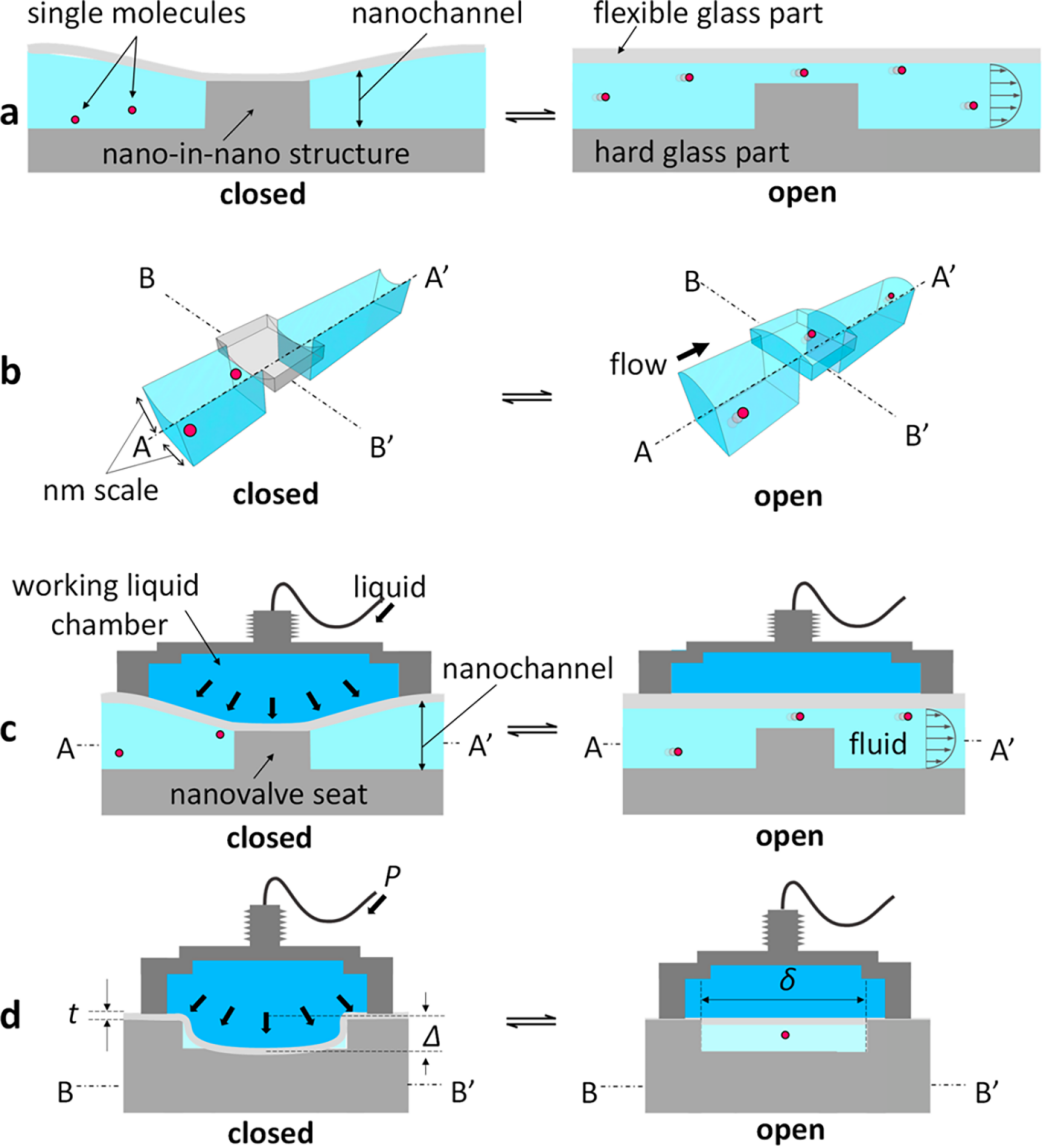
Concept of the study. (a, b) Conceptual drawings of controlling flows of single molecules using the flexible glass-based hybrid nanofluidic device. Longitudinal sections of the device along (c) the dotted lines A–A′ and (d) the dotted lines B–B′ in (b). The nanovalve part of the device is operated by actuating the deformation of the flexible glass sheet by applying liquid pressure to the working liquid chamber. In the closed state of the nanovalve, the flow of single small molecules in the nanochannel is blocked, whereas it is directed to pass the nanovalve part in the open state.

In principle, the maximum deformation of the flexible glass Δ under pressure *P* is described by the equation:^36^
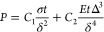
1where σ, *t*, and *E* denote the internal tensile stress, thickness, and Young’s modulus of flexible glass, respectively. δ is a parameter that depends on the shape of the flexible glass and is positively correlated with the width of the nanochannels. *C*_1_ and *C*_2_ are constants that can be determined from the Poisson’s ratio ν of the flexible glass.^36^ A large *P* is required to close the nanochannels by deformation of the flexible glass, owing to the ultrasmall width of the channels, which decreases δ. However, *P* is limited in practice to a maximum pressure that does not break the flexible glass. We selected a non-alkali ultrathin glass sheet (*t* = 4 μm, *C*_1_ = 0, *C*_2_ = 2.64, *E* = 73 GPa, and ν = 0.2) as the flexible glass in this study. Its ultrathin (small *t*) and flexible (small *E* and ν) properties are favorable to deformation in narrow nanochannels at a relatively small *P*, as revealed by the theoretically plotted relationship between Δ*a*nd *P*, shown in Figure S1.

To establish a proof of concept (Figure 1), a nanofluidic device with three sets of the same hybrid structure was designed (Figure 2a-d) and fabricated (Figure 2e-l). In each set of hybrid structures, all channel structures were fabricated on a hard glass (fused-silica) substrate. These channel structures constitute composite nanochannels connected to a pair of microfluidic channels (510 μm in width and 43 μm in depth) with inlets and outlets (500 μm in diameter) (Figure 2a, b). The channel radius can be expressed as the equivalent radius, i.e., *wd*/(*w* + *d*), where *w* and *d* are the width and depth of the channel, respectively.^16^ The composite nanochannels comprise two wide nanochannels (Figure 2b; 1.5 mm in length, 10 μm in width, 190 nm in depth; hereinafter called 1D-nanochannels), two narrow nanochannels (Figure 2c; 2.3 μm in length, 400 nm in width, 190 nm in depth, 129 nm in equivalent radius; hereinafter called 2D-nanochannels), and a nanovalve seat (Figure 2d; 750 nm in length, 750 nm in width, 143 nm in height). The nanovalve seat bridges the two 2D-nanochannels (Figure 2d), which further connect the two 1D-nanochannels (Figure 2c). In particular, the nanovalve seat (Figure 2d) is a delicate nano-in-nano structure that is essential for achieving the precise regulation of single-molecule flows in the nanochannel. It was fabricated (Figure 2f, g) using a nano-in-nano integration technology previously developed by us,^16,37,38,100^ which allows the precise fabrication of nanostructures inside tiny nanochannels.

**Figure 2:**
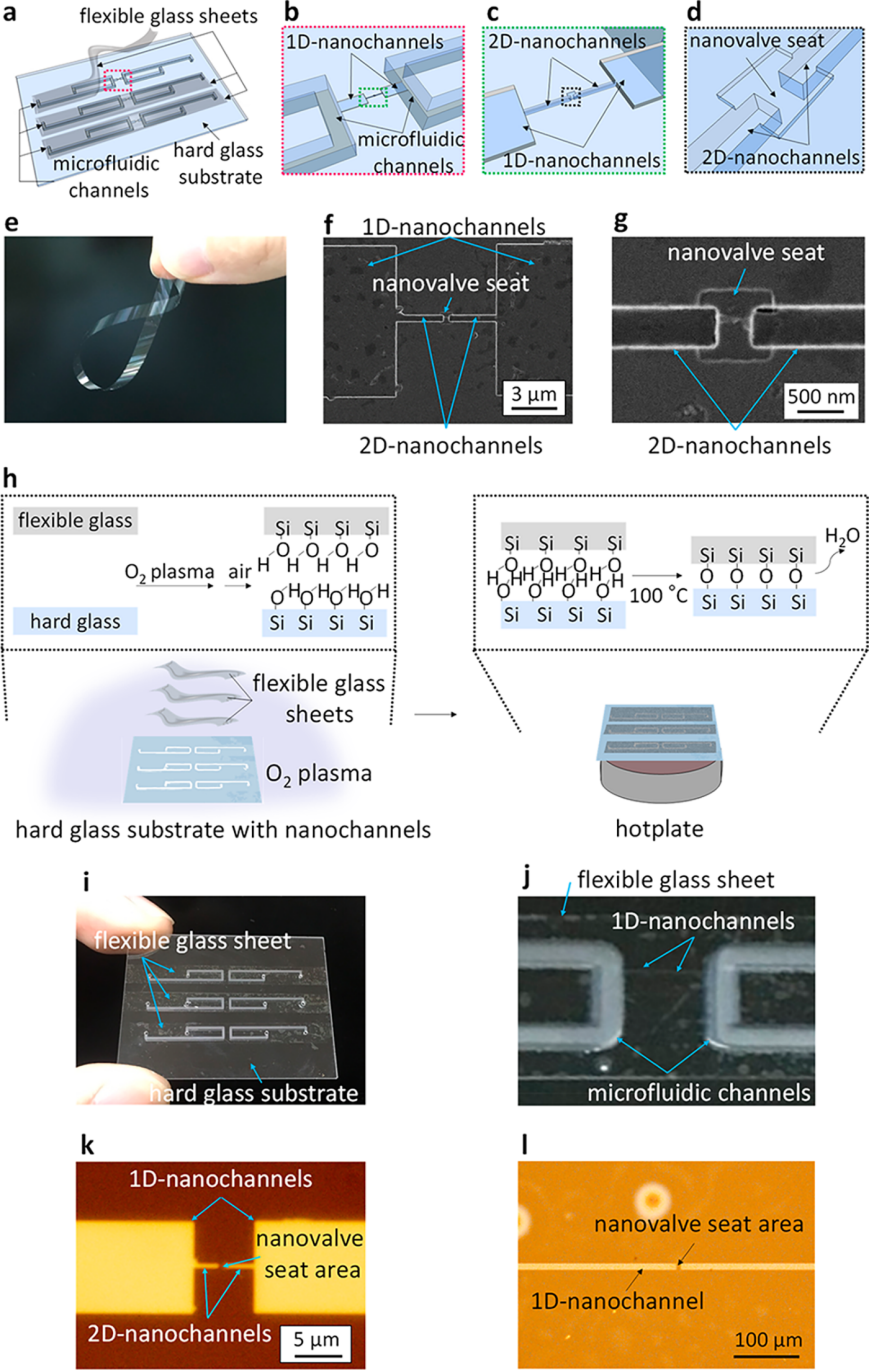
Fabrication and evaluation of the flexible glass-based hybrid nanofluidic device. Schematic images of (a) flexible glass-based hybrid nanofluidic device, (b) 1D-nanochannels between microfluidic channels, (c) 2D-nanochannels between 1D-nanochannels, and (d) the nanovalve seat between 2D-nanochannels. (e) Digital image of a flexible glass sheet. Scanning electron microscope images of (f) 2D-nanochannels between 1D-nanochannels and (g) the nanovalve seat between 2D-nanochannels. (h) Schematic images of the process for bonding flexible glass sheets and the hard glass substrate. (i) Digital images of the fabricated hybrid nanofluidic device. (j) Stereomicroscopic image exhibiting details of bonded area. Bright-field microscopic images of (k) the bonded channel structures and (l) details of the nanovalve seat area.

Generally, the bonding of dissimilar materials is a hurdle that impedes the development of hybrid devices. It also applies to our case. Despite the many methods available for bonding similar hard glass substrates,^39^ to the best of our knowledge, there are no reports on the bonding of dissimilar glass materials, such as fused-silica substrates and flexible glass sheets (Figure 2e) used in this study. Generally, thermal bonding and fusion bonding are used to bond hard-glass-based nanofluidic devices. These bonding methods usually require temperatures of 600 °C or higher.^16,37,38^ Inspired by our previous studies,^40,41^ we developed a high-pressure resistant, low-temperature (100 °C) bonding method (Figure 2h; Supplementary Note). This new method was used to bond the hybrid nanofluidic device (Figure 2i–l). Bonding enables the operation of the hybrid nanofluidic device at 200 kPa or less (Figure 3). In our experience, such high pressures suffice the requirement of most nanofluidic applications.

**Figure 3:**
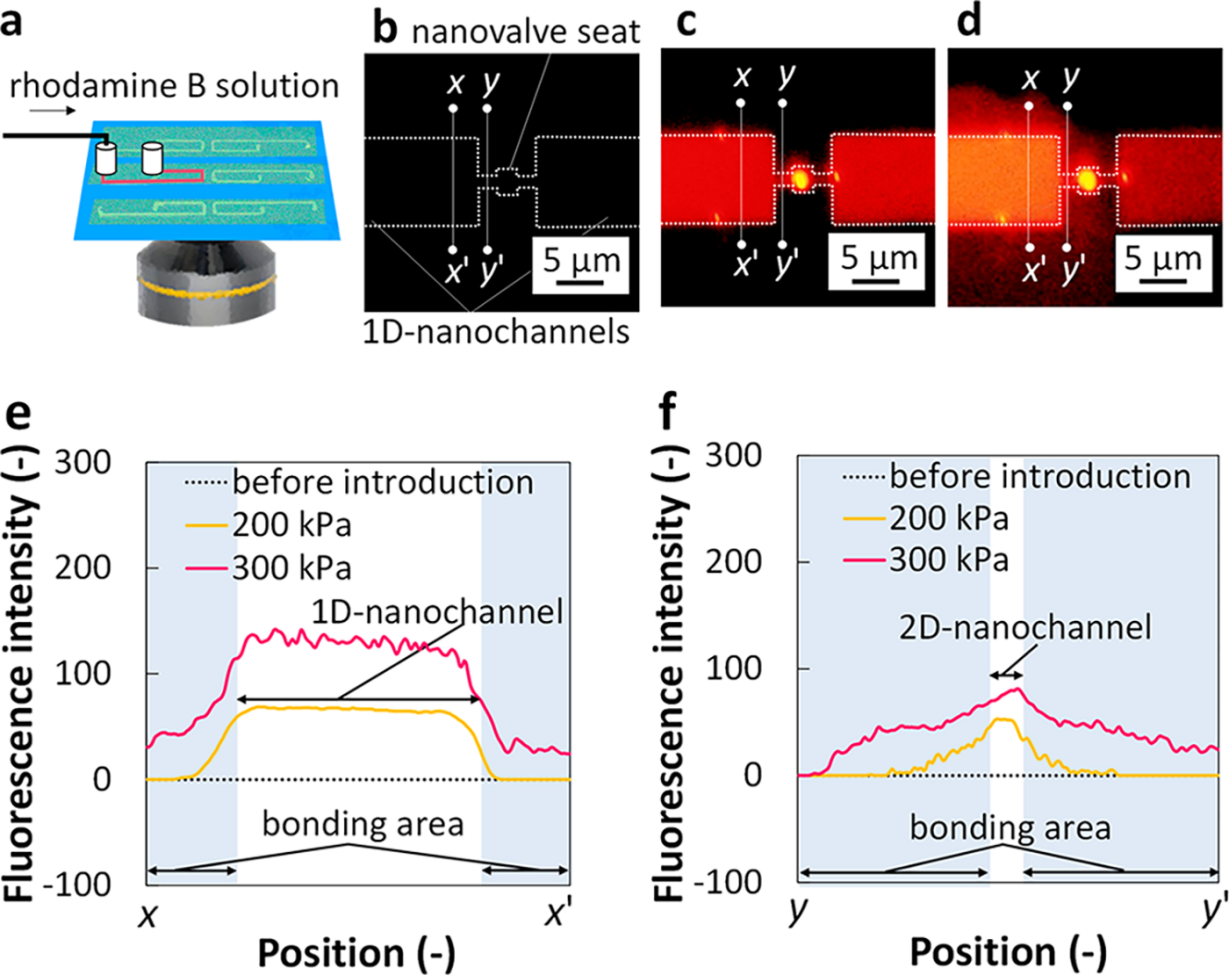
Evaluation of bonding. (a) Schematic image of the introduction of a solution of rhodamine B (100 μM) into the flexible glass-based hybrid nanofluidic device by applying SIP. Fluorescence images of the channel structure area (b) before and after the introduction of the solution under the SIP of (c) 200 kPa and (d) 300 kPa and line profiles of fluorescence intensities along lines (e) *x*–*x*′ and (f) *y*–*y*′ in (b–d), revealing that bonding enabled the operation of the hybrid nanofluidic device at 200 kPa or less but got leaked at 300 kPa.

The valving performance was evaluated according to the experimental protocol described in Figure 4a, b. The nanovalve was operated by actuating the deformation of the flexible glass sheet by applying liquid pressure (hereinafter called nanovalve actuation pressure, NCP) to the working liquid chamber (Figure 1c, d). First, ultrapure water was introduced into the nanofluidic device by capillary filling with the open nanovalve (without applying NCP). After the channels were filled with ultrapure water, the nanovalve was closed by applying an NCP of 700 kPa. In this step, no fluorescence was detected in any channel area (Figure 4c). Then, a fluorescent solution of rhodamine B (100 μM) was introduced into the hybrid nanofluidic device by air pressure (hereinafter called sample introduction pressure, SIP) at 200 kPa, using a custom-built liquid introduction system, as described elsewhere.^20,42,200^ During this step, no apparent fluorescence signal was detected in the nanochannel areas (Figure 4d, j), indicating no detectable net flow despite the high SIP. This result suggests that the nanovalve worked effectively in a closed state to resist high pressure. Theoretically, according to equation 1 and Figure S1, the deformation of the flexible glass sheet in the height direction is approximately 66 nm in the area of the nanovalve seat under an NCP of 700 kPa. Considering that the original space above the nanovalve seat is 47 nm, an NCP of 700 kPa could actuate sufficient nanoscale deformation for precisely blocking the 2D-nanochannels.

**Figure 4:**
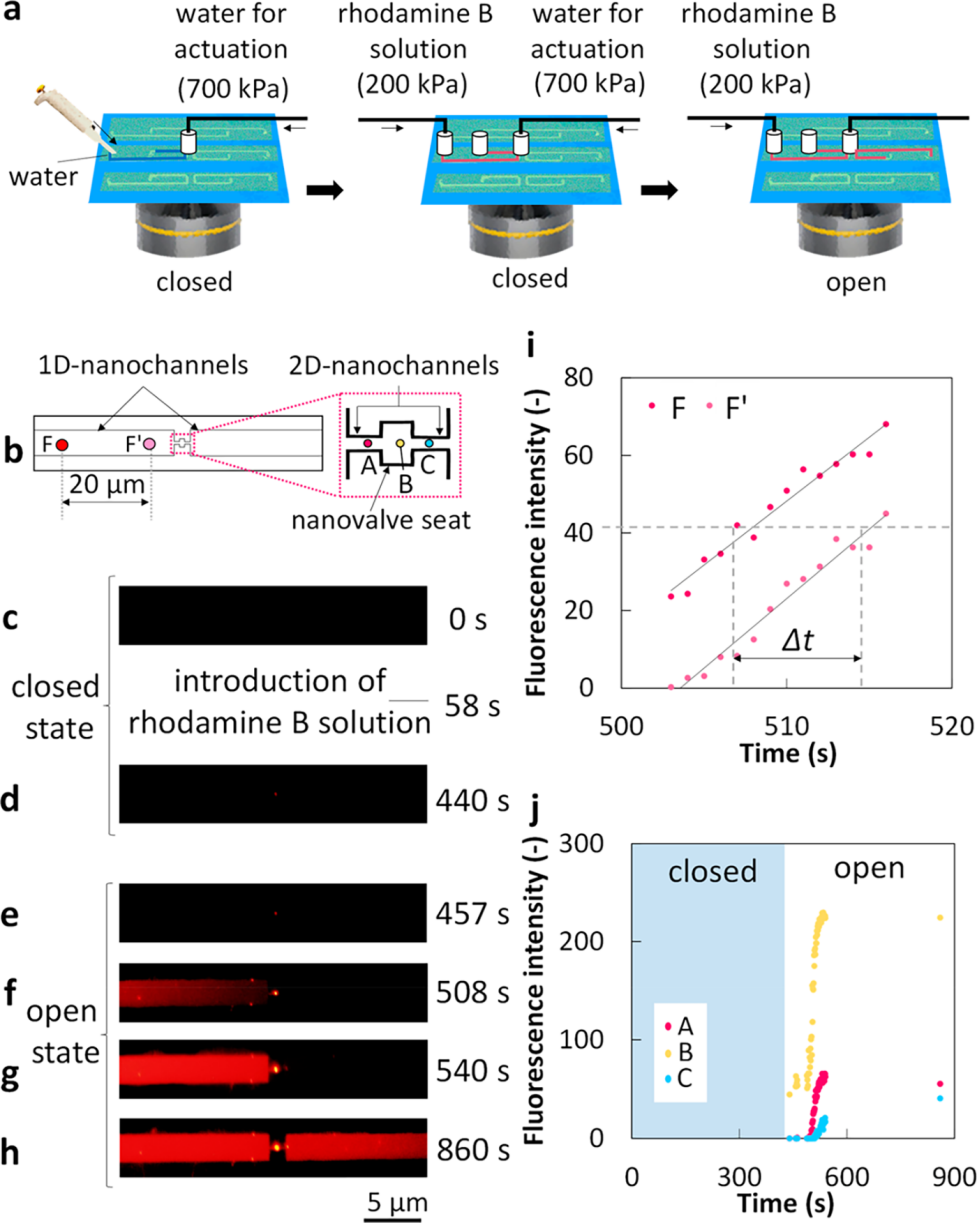
Verification and evaluation of valving performance. Schematic images of (a) the experimental protocol and (b) fluorescence microscopic observation area. Time-lapse fluorescence images of the observation area (c) before and after the introduction of a solution of rhodamine B, (d) during the closed state of the nanovalve, and (c–h) during the open state of the nanovalve. (i) Change in fluorescence intensity over time at locations F and F′ in (b). (j) Change in fluorescence intensity over time at locations A, B, and C in (b).

Subsequently, the NCP was relieved to switch the nanovalve to the open state. Presently, apparent fluorescence was observed in the channel area on the left side of the nanovalve seat (Figure 4e, f). As the fluorescence in the channel area on the left became stronger (Figure 4f–h), fluorescence appeared in the channel area on the right side of the nanovalve seat (Figure 4f–g) and it finally reached almost the same level as that on the left side (Figure 4h). These imaging results revealed that after relieving the NCP, the deformation of the flexible glass sheet was effectively recovered, and the nanovalve was successfully opened.

The changes in fluorescence profiles at different locations in the 1D-nanochannels further clarify the speed of the actuated nanofluidic flows. Real-time fluorescence intensities at two locations 20 μm apart in 1D-nanochannels (F and F′ in Figure 4b) are plotted in Figure 4i. By comparing the plots during a period of significant change in fluorescence intensity, the speed of the nanofluidic flow in the 1D-nanochannel was measured as 2.8 μm s^–1^ from the average time (Δ*t*) between each line at the same fluorescence intensity (i.e., 503–516 s in Figure 4i). Based on this measured speed, the speeds of nanofluidic flows in the 2D-nanochannel and the nanovalve seat area were calculated to be 70 μm s^–1^ and 151 μm s^–1^, respectively.

The real-time profiles (Figure 4j) of the fluorescence intensity in front of (point “A” in the Figure 4b), at (point “B” in the Figure 4b), and behind (point “C” in the Figure 4b) the nanovalve seat during imaging provide more detail about the nanofluidic flow around the open nanovalve. The fluorescence intensities at all three points briefly increased significantly when the valve was opened, and finally saturated to steady-state conditions (Figure 4j). Although there is initially an apparent lag in the change in the fluorescence intensity between points A and C, the lag becomes very small at the end of the experiment. This result suggests that a net flow in the nanochannels was induced and continuously driven by the SIP after the nanovalve was opened.

In addition, it is worth noting that the nanovalve seat area (point “B”) exhibited remarkably higher fluorescence intensity than the 2D nanofluidic channels (points “A” and “C”) in the steady-state condition (Figure 4j). Similar phenomenon was also observed in the bonding performance evaluation experiment of the hybrid device, as shown in Figure 3c, d. This phenomenon is quite intriguing; the nanovalve seat area was supposed to exhibit a lower intensity of fluorescence than the 2D nanofluidic channels, considering that the absolute number of fluorescent molecules in the nanovalve seat area is significantly less than those in the 2D nanofluidic channels; this conjecture is based on the fact that the space (or volume) between the flexible glass sheet and the top of the nanovalve seat is significantly smaller than the 2D nanofluidic channels; however, the experimental result is quite different from the conjecture. This intriguing phenomenon is possibly ascribed to a signal amplification resulting from the nanoconfinement effects in the extremely small nanofluidic space (47 nm in depth) formed in the nanovalve seat area. Although the mechanism is unclear and needs to be elucidated in the future, a similar phenomenon has been reported in some other electrochemical analysis and sensing systems with nanoconfined structures.^43^ Such signal amplification mechanism in the confined ultrasmall nanofluidic space is very favorable for the development of ultrasensitive detection using an ordinary fluorescence microscopy setup rather than the expensive and specific high-spec microscopy systems (e.g., total internal reflection fluorescence microscope, confocal fluorescence microscope, super-resolution fluorescence microscopy), which are indispensable in the field of ultrasensitive detection.

The direct regulation of the transport of single molecules in nanofluidic flows using the device was further demonstrated using a solution of sulfo-cyanine 3 carboxylic acid (Cy3; Ex/Em = 555 nm/569 nm) at 200 nM. This concentration confines discrete single molecules in the nanochannels with picoliter (10^–12^ L, pL) to attoliter (10^–18^ L, aL) volumes, thereby forming single-molecule flows. The Cy3 solution was introduced into the hybrid nanofluidic device via capillary filling in the closed nanovalve. The nanochannel area, as shown in Figure 5a, was observed using an ordinary fluorescence microscope with a laser (532 nm, 50 mW) as the excitation light source (Figure S2). Dot-like fluorescence signals exhibiting fluorescence blinking were detected in the nanochannels on the left (Figure 5b, c; Supplementary Movie 1). Fluorescence blinking, or the so-called fluorescence intermittency, is an intriguing single-molecule random switching between on (bright) and off (dark) of an individual fluorophore during its continuous excitation.^44,45^ Fluorescence blinking has been widely used in the identification of single molecules and the analysis of single-molecule dynamics.^46^ Cy3 is one of the most common fluorescent molecules of choice, owing to its properties such as fluorescence blinking with a period lasting seconds.^47,48^ In addition, although the aforementioned, expensive high-spec microscopy systems are indispensable in the field of ultrasensitive detection, our result (Figure 5b, c) reveals that owing to the nanoconfinement effect of the ultrasmall nanofluidic space, the coupling of the hybrid device and ordinary fluorescence microscopy provides a simple experimental setup for achieving ultrasensitive detection and the tracking of single small molecules in fluids. Moreover, no signals were detected in the nanochannels on the right side of the nanovalve seat, suggesting that the closed-state nanovalve can dam single-molecule flow.

**Figure 5:**
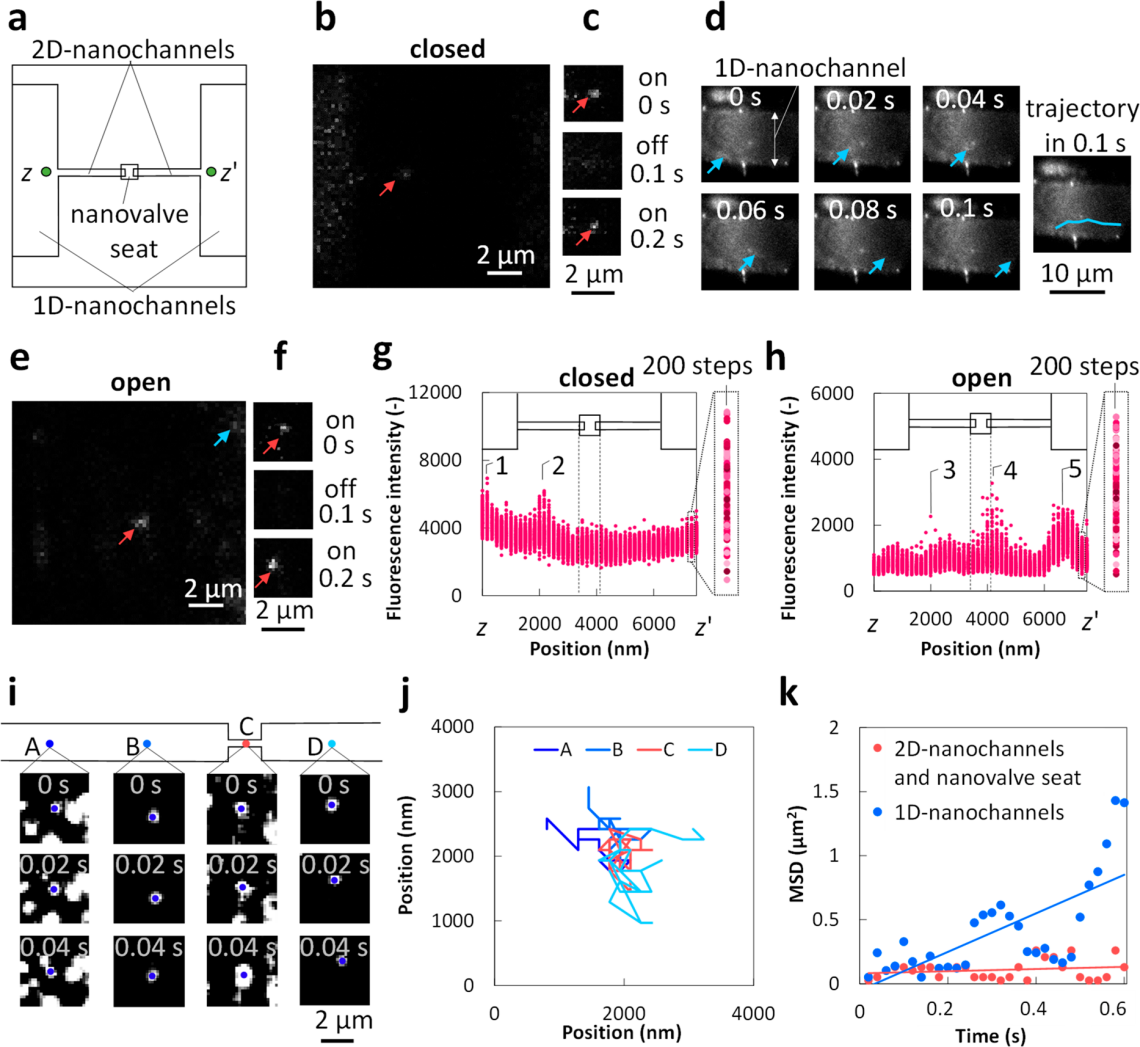
Active regulation of flows of single small molecules and dynamic behaviors of single molecules confined in nanofluidic conditions. (a) Schematic image of fluorescence microscopic observation area. (b) A fluorescence image of a single Cy3 molecule in the nanochannel with the closed nanovalve and (c) time-lapse fluorescence images exhibiting its blinking. (d) Fluorescence images and trajectories of the flow of a single Cy3 molecule in 1D-nanochannels with the nanovalve in the open state. (e) A fluorescence image of three single Cy3 molecules confined in nanochannels and (f) time-lapse fluorescence images exhibiting the blinking of a single Cy3 molecule, indicated by the red arrow in e with the nanovalve in the open state. (g) Real-time fluorescence profiles of the fluorescence intensity over 200 consecutive frames along a line between *z*–*z*′ shown in (a) under the same condition as that in (b) (nanovalve in the closed state). (h) Real-time fluorescence profiles of the fluorescence intensity over 200 consecutive frames along a line between *z*–*z*′ shown in (a) under the same condition as that in (e) (nanovalve in the open state). (i) Time-lapse fluorescence images and (j) trajectories of single Cy3 molecules at different locations of A, B, C, and D in nanochannels under the same condition as that in (e); blue dots in (i) show the centers of tracked single molecules. (k) Change in mean-squared displacements (MSDs) of single Cy3 molecules confined in different nanofluidic areas with different spatial restrictions under the same condition as that in (e).

Figure 5d shows time-lapse images and the tracking trajectory of a single Cy3 molecule flowing by capillary filling in the 1D-nanochannel on the right side with the nanovalve in the open state (Supplementary Movie 2). The discrete single Cy3 molecules confined in the nanochannels are directed by capillary action to form a continuous flow along the nanochannel, as indicated by real-time single-molecule tracking. In addition, the speed of a single molecule moving in the flow direction was 120 μm s^–1^, as revealed by a calculation based on the trajectory. Figure 5e shows an image of single-molecule tracking after capillary action had completely diminished. Fluorescence signals from the blinking (Figure 5f and Supplementary Movie 3) of single Cy3 molecules engaged in Brownian motions (as revealed in Figure 5i–k) were detected on both sides of the nanovalve seat. This result implies that single Cy3 molecules flowed from the nanochannel on the left to the nanochannel on the right through the nanovalve seat.

Furthermore, real-time fluorescence profiles of the fluorescence intensity over 200 consecutive frames along a line between *z*–*z*′ as shown in Figure 5a were plotted (Figure 5g, h), and correspond to real-time observations under the same conditions as those in Figure 5b (nanovalve in the closed state) and Figure 5e (nanovalve in the open state). Because the *z*–*z*′ line moves horizontally through the 1D and 2D-nanochannels on both sides and through the central nanovalve seat area, the profiles provide details about single-molecule events that occur in the hybrid nanofluidic device. In both Figure 5g and 5h, there are specific locations exhibiting significantly increased fluorescence intensity (peaks 1–5), ascribed to the occurrence of single-molecule events (i.e., Brownian motion, as shown in Figure 5i–k) at those locations. With the nanovalve closed, single-molecule events appeared only in the nanochannel on the left side of the nanovalve seat (peaks 1 and 2 in Figure 5g), further revealing that the closed nanovalve blocked the passage, thereby preventing single molecules from flowing to the opposite side. In contrast, with an open nanovalve, such single-molecule events were observed in the nanochannels on both sides (peaks 3 and 5 in Figure 5h) and in the nanovalve seat area (peak 4 in Figure 5h), further suggesting that the passage recovered after the nanovalve was opened. These results further support that the hybrid nanofluidic device possesses the capability to actively regulate the flow of single molecules. In addition, it is worth noting that among the peaks in Figure 5h, peak 4, which corresponds to the nanovalve seat, contains single-molecule events with significantly higher fluorescence intensities than the other peaks (3 and 5). Similar to the intriguing phenomenon afore-discussed according to Figure 4j, this phenomenon could be ascribed to signal amplification resulting from the nanoconfinement effects in an extremely small nanofluidic space formed in the nanovalve seat area.

To further clarify the behavior of the single molecules confined in the nanochannels, the obtained single-molecule motion in the area of the 1D-nanochannels (locations A, B, and D in Figure 5i) and the area of the 2D-nanochannels and nanovalve seat (location C in Figure 5i) under the same conditions as those in Figure 5e were analyzed. Figure 5i and 5j displays three consecutive frames of single-molecule fluorescence images and the trajectories of single molecules during 31 consecutive frames at designated locations, respectively. No fixed direction was observed in any of the trajectories of the single molecules at the designated locations, revealing that these single molecules were performing Brownian motions because of the absence of directed net flow. In addition, the single molecules confined in the areas of the 1D-nanochannels were able to randomly migrate more widely than those confined in the areas of the 2D-nanochannels and the nanovalve seat, as indicated by Figure 5j. This difference between the 1D and 2D-nanochannels to confine single molecules was further investigated by analyzing the time dependency of the mean square displacement (MSD) of the single molecules (Figure 5k). MSD is a measure of the deviation of the position of a molecule (or a particle) with respect to a reference position over time.^49^ It is the most common measure of the spatial extent of Brownian motion. As shown in Figure 5k, an approximate curve with a larger slope was confirmed for the 1D-nanochannels than for the 2D-nanochannels and nanovalve seat. The local diffusion coefficient calculated as one-fourth of the slope by the Einstein relation^50^ for two-dimensional tracking is 7.5 × 10^–2^ μm^2^ s^–1^ for the Cy3 molecules confined in the 1D-nanochannels, and it is 4.5 × 10^–3^ μm^2^ s^–1^ for the Cy3 molecules confined in the area of 2D-nanochannels and the nanovalve seat. The diffusion coefficient measures the ability of a molecule to diffuse through a medium. Hence, this result implies that the nanospace of the 2D-nanochannel and the nanovalve seat exhibited stronger restriction of the Brownian motion of single small Cy3 molecules than the 1D-nanochannels. This difference is ascribed to the 1D-nanochannel providing only a one-dimensional spatial restriction, whereas the 2D-nanochannel and the nanovalve seat area offer a two-dimensional spatial restriction for a small, confined molecule. These characteristics of the hybrid nanofluidic device are favorable for single-molecule studies and applications because these simultaneously enable varied well-defined nanoconfinement environments with actively regulable nanofluidic conditions in a single platform.

In conclusion, we developed a hybrid nanofluidic device with an in situ nanofluidic valving mechanism capable of effectively translating a reversible nanometer-scale mechanical deformation into a local, direct, and active valving function that enables the pinpoint blockage and the direction of flow of single small molecules confined in tiny nanochannels. The use of the device also allows for the sensitive detection and real-time tracking of regulated single small molecules in nanofluidic conditions using a simple ordinary fluorescence microscopy setup. Therefore, the dynamic behavior of single molecules confined under different nanofluidic conditions with different spatial restrictions was further elucidated. This device and method can offer a platform and mechanism for conducting single-molecule studies in actively regulated fluidic conditions, which is favorable for understanding the original behavior of individual molecules in their natural forms. In addition, our device and approach introduce the possibility of handling single small molecules in a solution or fluid. Our study is a first step toward manipulating individual small molecules in solution freely, which is one of the ultimate goals of chemists and biologists. In the future, realizing such manipulation will open avenues for performing processes involved in chemical and biological interactions and reactions in single-molecule units (namely, single-molecule regulated chemical and biological processes), thus offering novel mechanisms, powerful tools, and impactful applications that could revolutionize chemistry, biology, and materials science, and finally transform the industry.

## Supporting Information Available

Methods section, Figure S1–S2, and Supplementary note: device fabrication; liquid introduction and handling; nanovalve operation; microscopic imaging setup, data acquisition, and analysis; relation between pressure *P* and maximum deformation of flexible glass Δ on the nanovalve seat; optical setup for observation of single molecules by laser excitation; bonding and evaluation (PDF)
Fluorescence blinking of a single Cy3 molecule in the nanochannel under the closed state of the nanovalve, corresponding to Figure 5c (AVI)
Flow of a single Cy3 molecule in 1D-nanochannels under the open state of the nanovalve, corresponding to Figure 5d (AVI)
Fluorescence blinking of the single Cy3 molecule indicated by the red arrow in Figure 5e under the open state of the nanovalve (AVI)

## Supplementary Material

nl2c04807_si_001.pdf

nl2c04807_si_002.avi

nl2c04807_si_003.avi

nl2c04807_si_004.avi
