# Supplementary material for: Flexible Glass-Based Hybrid Nanofluidic Device to Enable the Active Regulation of Single-Molecule Flows
Source: Nano Lett. 2023 Mar 7;23(6):2210–8. doi: 10.1021/acs.nanolett.2c04807 (PMC10804405; doi:10.1021/acs.nanolett.2c04807)
Supplement: Supplementary file 1 — nl2c04807_si_001.pdf [file nl2c04807_si_001.pdf]

(Cite the paper: https://doi.org/10.1021/acs.nanolett.2c04807)

Supporting Information

Flexible glass-based hybrid nanofluidic device to enable the active regulation of single-molecule flows

*Hiroto Kawagishi,[1,2] Shun-ichi Funano,[3] Yo Tanaka[3] and Yan Xu[1,2,4,5]**

[1] Department of Chemical Engineering, Graduate School of Engineering, Osaka Metropolitan University, 1-2, Gakuen-cho, Naka-ku, Sakai, Osaka 599-8570, Japan

[2] Department of Chemical Engineering, Graduate School of Engineering, Osaka Prefecture University, 1-2, Gakuen-cho, Naka-ku, Sakai, Osaka 599-8570, Japan

[3] Center for Biosystems Dynamics Research, RIKEN, 1-3 Yamadaoka, Suita, Osaka 565-0871, Japan

[4] Japan Science and Technology Agency (JST), PRESTO, 4-1-8 Honcho, Kawaguchi, Saitama 332-0012, Japan

[5] Japan Science and Technology Agency (JST), CREST, 4-1-8 Honcho, Kawaguchi, Saitama 332-0012, Japan

[*] Corresponding author: (Y. Xu) E-mail: xuy@omu.ac.jp

Supplementary movie 1: Fluorescence blinking of a single Cy3 molecule in the nanochannel under the closed state of the nanovalve, corresponding to Figure 5c.

Supplementary movie 2: Flow of a single Cy3 molecule in 1D-nanochannels under the open state of the nanovalve, corresponding to Figure 5d.

Supplementary movie 3: Fluorescence blinking of the single Cy3 molecule indicated by the red arrow in Figure 5e under the open state of the nanovalve.

METHODS

Device fabrication

Nanochannel structures with nanovalve seats were fabricated on a hard glass substrate (fused silica; 40 mm × 30 mm, 0.7 mm thick; Sendai Quartz, Japan) according to a Nano-in-Nano integration process[1–4] by the combination of electron beam lithography using an electron beam exposure system (ELS-7500EXI, ELIONIX, Japan) and plasma dry etching using a plasma etching system (RIE-10NR, SUMCO, Japan).[5,6] Microfluidic channels were fabricated on the same hard glass substrate using a milling machine (KitMill RD300, ORIGINALMIND, Japan). Holes of inlets and outlets for liquid introduction were fabricated on flexible glass sheets (40 mm × 5 mm, 4 μm thick; OA-10G, non-alkali glass, Nippon Electric Glass, Japan) by punching with an injection needle. After the microfabrication, surfaces of the hard glass substrates and flexible glass sheets were perfectly cleaned and then treated by an O_2 plasma process using a plasma modifier (PM 100, Yamato Scientific, Japan) for 5 min. Subsequently, the flexible glass sheet was carefully placed to the channels on the hard glass substrate using tweezers to achieve a preliminary bonding, followed by a thermal treatment on a hot plate (HHP-441V, AS ONE, Japan) at 100 °C for 10 min to achieve the final bonding.

Liquid introduction and handling

All sample liquids were filtered using 200 nm filters (Minisart, Sartorius Stedim Biotech, France) before introducing into the nanofluidic device. As described in the results section, either a pressure driven flow mechanism or a capillary filling flow mechanism was adopted to introduce sample liquids into the nanochannels according to different purposes of experiments. Both these are the most used liquid handling mechanisms in the field of nanofluidics. For the pressure driven flow mechanism, sample liquids were introduced using a custom-built air pressure-based liquid

introduction system according to a protocol described previously.[2,7,8] In this mechanism, the air pressure (i.e., SIP) can be precisely controlled by a high-precision pressure controller (OB1 MK3+, Elve Flow, France), thereby, reproducible pressure-driven flows could be generated in the nanofluidic device. In addition, the same air pressure-based liquid introduction system was used to generate the liquid pressure (i.e., NCP) to actuate the deformation of the flexible glass sheet in the operation of the nanovalve. For the capillary filling flow mechanism, sample liquids were simply introduced from inlets of the nanofluidic device by using a micropipette by taking advantage of the capillary action of the microfluidic and nanochannels whose wall surfaces were hydrophilic.

Nanovalve operation

In the experiment, the nanovalve was operated by actuating the deformation of the flexible glass sheet via applying a liquid pressure (i.e., NCP) to the working liquid chamber (Figure 1c, d), which was built directly on the flexible glass sheet by taking advantage of the small inner space of a custom-made Teflon connector (500 μm i.d.) with high liquid tightness (Figure 4a). The working liquid chamber was deliberately located on the specific position of the upper flexible glass sheet corresponding to the position of the nanovalve seat in the lower hard glass substrate (Figure 1c, d), guaranteeing that the deformation of the flexible glass could be precisely and effectively translated into the function of the nanovalve.

Microscopic imaging setup, data acquisition, and analysis

The scanning electron microscopy images of nanochannels and nanovalve seats were obtained using a field emission scanning electron microscope (FE-SEM; SU8010, Hitachi High-Tech, Japan). Two types of fluorescence microscopic imaging setups were used for optical imaging according to different purposes of experiments. Except for single-molecule imaging, an upright

fluorescence microscope (BX53, Olympus, Japan) with a CCD camera (DP73, Olympus, Japan) and a mercury lamp for fluorescence excitation were used. For single molecule experiments, an optical setup comprising inverted fluorescence microscopy (IX71, Olympus, Japan), an EMCCD camera (iXon-Ultra-888, Andor, UK), and a continuous wave (CW) laser (532 nm, 50 mW, CivilLaser, China) was used, as described in Figure S2. The microscope was installed with an objective lens (LUCPlanFLN 60X, Olympus, Japan), a dichroic mirror (Di02-R532-25x36, Semrock, USA), and an emission filter (BLP01-532R-25, Semrock, USA) for fluorescence detection and observation. The laser was introduced into the microscope through a beam expander (Sigmakoki, Japan), convex lens (Sigmakoki, Japan), and Iris diaphragm (Sigmakoki, Japan) for fluorescence excitation. Obtained microscopic images were analyzed using ImageJ software (NIH, Bethesda, MD, USA).

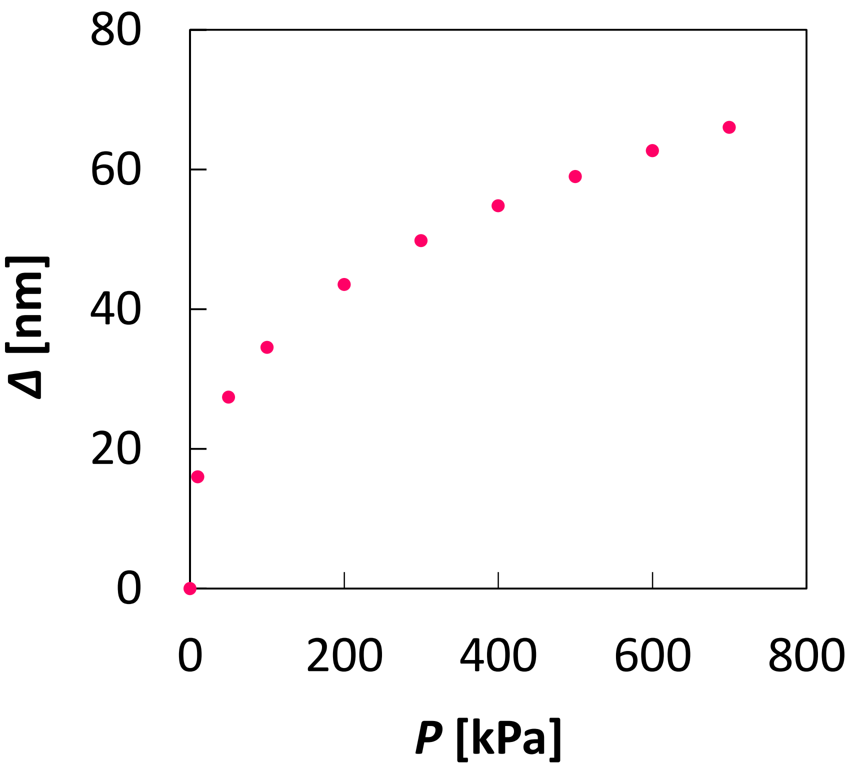

Figure S1. Relation between pressure P and maximum deformation of flexible glass Δ on the nanovalve seat.

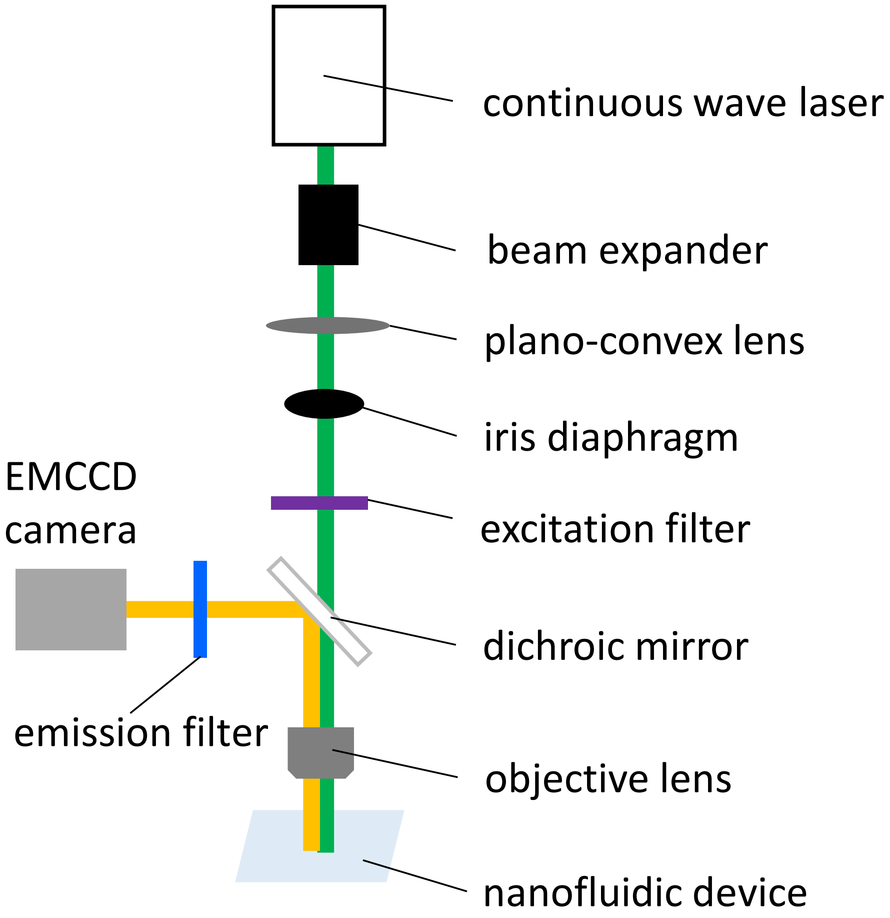

Figure S2. Schematic image of optical setup for observation of single molecules by laser excitation.

Supplementary Note: Bonding and evaluation

Generally, thermal bonding and fusion bonding are major bonding methods for hard glass-based nanofluidic devices. These major bonding methods usually require a high temperature of 600 °C or higher.[1–3] Hence, we first tested the thermal bonding of the hard glass substrate and the flexible glass sheet at 600 °C. As a result, however, although the partial area was well bonded, cracks were observed in the flexible glass sheet (Supplementary Figure 1). Such cracking is possibly ascribed to the large expansion difference between the flexible glass sheet (thermal expansion coefficient $= 38 \times 10^{-7}$ K^{-1}) and the hard glass substrate (thermal expansion coefficient $= 5.5 \times 10^{-7}$ K^{-1}) at 600 °C. To avoid cracking, a low temperature bonding method at 100 °C was explored (Figure 2h). The method was originally inspired by our previous studies,[5,6] in which low-temperature (below 200 °C) bonding methods for conventional glass nanofluidic devices were developed based on either a two-step sequential (i.e., O_2 and N_2) plasma surface activation process[5] or a one-step surface activation using an O_2/CF_4 plasma treatment.[6] In this study, rather than the use of the plasma of multiple types of gases, a modified process using only O_2 plasma was explored (Figure 2h). The O_2 plasma treatment helps to activate both types of glasses by generating hydrophilic silanol groups (Si-OH) on the surface of the glasses, which is very favorable for bonding. After the O_2 plasma activation, the activated surfaces of the flexible glass sheet and the hard glass substrate were immediately contacted with each other and heated at 100 °C to induce covalent bonds (Si-O-Si) between the silanol groups on both surfaces of the glasses (Figure 2h). As a result, the flexible glass sheets were successfully bonded to the hard glass substrate without cracking (Figure 2i-l).

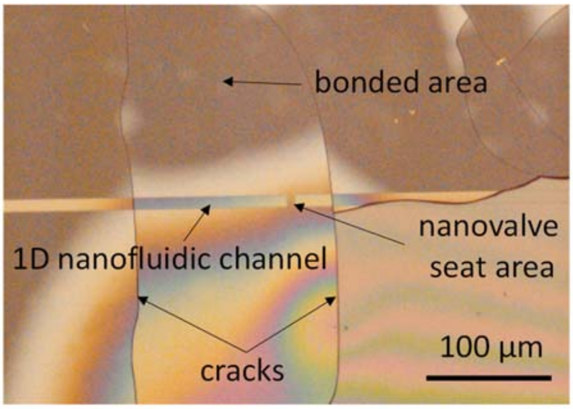

Supplementary Figure 1. *Microscopic image of 1D-nanochannels in the nanofluidic device by bonding at 600 °C.*

To further evaluate the performance of the bonding, a fluorescent solution of rhodamine B (100 μM) was introduced into the nanochannels by air pressure (i.e.,, SIP) (Figure 3a) using a custom-built liquid introduction system as described elsewhere.[4,8] Before introduction, no fluorescence was detected (Figure 3b). After the introduction at 200 kPa, strong fluorescence of rhodamine B was observed on both sides of 1D and 2D-nanochannels (Figure 3c, e, and f), revealing that in the open state of the nanovalve the solution was directed into the nanochannels. In particular, no fluorescence was observed in the bonded area (non-channel area), highlighting no detectable leakage in the bonded hybrid nanofluidic device under the SIP of 200 kPa. In contrast, after introduction at 300 kPa, obvious fluorescence was observed in the originally bonded area (non-channel area) (Figure 3d, e, and f), indicating leakage occurrence. These results suggest that the newly-developed bonding method enables the operation of the hybrid nanofluidic device at 200 kPa or less.

REFERENCES

(1) Xu, Y.; Matsumoto, N.; Wu, Q.; Shimatani, Y.; Kawata, H. Site-Specific Nanopatterning of Functional Metallic and Molecular Arbitrary Features in Nanofluidic Channels. *Lab Chip* **2015**, *15* (9), 1989–1993.

(2) Xu, Y.; Xu, B. An Integrated Glass Nanofluidic Device Enabling In-Situ Electrokinetic Probing of Water Confined in a Single Nanochannel under Pressure-Driven Flow Conditions. *Small* **2015**, *11* (46), 6165–6171.

(3) Kamai, H.; Xu, Y. Fabrication of Ultranarrow Nanochannels with Ultrasmall Nanocomponents in Glass Substrates. *Micromachines* **2021**, *12* (7), 775.

(4) Kawagishi, H.; Kawamata, S.; Xu, Y. Fabrication of Nanoscale Gas–Liquid Interfaces in Hydrophilic/Hydrophobic Nanopatterned Nanofluidic. *Nano Lett.* **2021**, *21* (24), 10555–10561.

(5) Xu, Y.; Wang, C.; Dong, Y.; Li, L.; Jang, K.; Mawatari, K.; Suga, T.; Kitamori, T. Low-Temperature Direct Bonding of Glass Nanofluidic Chips Using a Two-Step Plasma Surface Activation Process. *Anal. Bioanal. Chem.* **2012**, *402* (3), 1011–1018.

(6) Xu, Y.; Wang, C.; Li, L.; Matsumoto, N.; Jang, K.; Dong, Y.; Mawatari, K.; Suga, T.; Kitamori, T. Bonding of Glass Nanofluidic Chips at Room Temperature by a One-Step Surface Activation Using an O2/CF4 Plasma Treatment. *Lab Chip* **2013**, *13* (6), 1048–1052.

(7) Xu, Y.; Shinomiya, M.; Harada, A. Soft Matter-Regulated Active Nanovalves Locally Self-Assembled in Femtoliter Nanofluidic Channels. *Adv. Mater.* **2016**, *28* (11), 2209–2216.

(8) Fukuda, S.; Xu, Y. A Biomimetic Anti-Biofouling Coating in Nanofluidic Channels. *J. Mater. Chem. B* **2022**, *10* (14), 2481–2489.
